# Chlorpromazine Increases the Expression of Polysialic Acid (PolySia) in Human Neuroblastoma Cells and Mouse Prefrontal Cortex

**DOI:** 10.3390/ijms18061123

**Published:** 2017-05-24

**Authors:** Chikara Abe, Saki Nishimura, Airi Mori, Yuki Niimi, Yi Yang, Masaya Hane, Ken Kitajima, Chihiro Sato

**Affiliations:** 1 Bioscience and Biotechnology Center, Nagoya University, Nagoya 464-8601, Japan; abe.chikara@f.mbox.nagoya-u.ac.jp (C.A.); kenk@rj9.so-net.ne.jp (S.N.); mori.airi@d.mbox.nagoya-u.ac.jp (A.M.); the.one.p21@gmail.com (Y.N.); yang.yi@j.mbox.nagoya-u.ac.jp (Y.Y.); hane.masaya@gmail.com (M.H.); kitajima@agr.nagoya-u.ac.jp (K.K.); 2 Graduate School of Bioagricultural Sciences, Nagoya University, Nagoya 464-8601, Japan

**Keywords:** polysialic acid, neural cell adhesion molecule, schizophrenia, polysialyltransferase, brain

## Abstract

The neural cell adhesion molecule (NCAM) is modified by polysialic acid (polySia or PSA) in embryonic brains. In adult brains, polySia modification of NCAM is only observed in restricted areas where neural plasticity, remodeling of neural connections, or neural generation is ongoing although the amount of NCAM remains unchanged. Impairments of the polySia-expression and several single nucleotide polymorphisms (SNPs) of the polysialyltransferase (polyST) ST8SIA2 gene are reported to be associated with schizophrenia and bipolar disorder. Chlorpromazine (CPZ) is well-known as an agent for treating schizophrenia, and our hypothesis is that CPZ may affect the polySia expression or the gene expression of polySTs or NCAM. To test this hypothesis, we analyzed the effects of CPZ on the expression of polySia-NCAM on human neuroblastoma cell line, IMR-32 cells, by immunochemical and chemical methods. Interestingly, the cell surface expression of polySia, especially those with lower chain lengths, was significantly increased on the CPZ-treated cells, while mRNAs for polySTs and NCAM, and the amounts of total polySia-NCAM remained unchanged. The addition of brefeldin A, an inhibitor of endocytosis, suppressed the CPZ-induced cell surface polySia expression. In addition, polySia-NCAM was also observed in the vesicle compartment inside the cell. All these data suggest that the level of cell surface expression of polySia in IMR-32 is highly regulated and that CPZ changes the rate of the recycling of polySia-NCAM, leading to the up-regulation of polySia-NCAM on the cell surface. We also analyzed the effect of CPZ on polySia-expression in various brain regions in adult mice and found that CPZ only influenced the total amounts of polySia-NCAM in prefrontal cortex. These results suggest a brain-region-specific effect of CPZ on the expression of total polySia in mouse brain. Collectively, anti-schizophrenia agent CPZ consistently up-regulates the expression polySia at both cellular and animal levels.

## 1. Introduction

The polysialic acid (polySia/PSA) is a linear homopolymer of the sialic acid (Sia) with the degree of polymerization (DP) that varies from 8 to 400 Sia residues [1,2]. The majority of polySia is expressed on the neural cell adhesion molecule (NCAM) in embryonic brain, while it almost disappears in the adult brain. However, the expression of polySia-NCAM persists in restricted areas of the adult brain such as the olfactory bulb (OB) and hippocampus (HIP), which are both known for their plasticity [1,2,3]. PolySia on NCAM is considered as an anti-adhesive molecule that repulses cell-cell interactions due to its physical properties [4]. Through its anti-adhesive effects on cell-cell interactions, polySia has been considered to be involved in the regulation of neuronal functions [3]. Recently, we have demonstrated that polySia functions not only as an anti-cell adhesion molecule but also as a reservoir scaffold for various neurological active molecules, such as brain derived neurotrophic factor (BDNF) [5,6,7], dopamine [8], and fibroblast growth factor (FGF2) [6,9]. Thus, we have hypothesized that polySia expression on the cell surface might be highly regulated for normal brain functioning via displaying highly regulated repulsive and attractive fields [1]. Indeed, there have been several reports on the abnormal expression of polySia in diseases such as schizophrenia [10,11] and various cancers [2]. In particular, the polySia biosynthesizing enzyme, ST8SIA2 (STX, siat8b), has been reported to have some association with schizophrenia [8,12,13], bipolar disorder [14], and small cell lung cancer [15].

Schizophrenia (SZ) is a one of the most severe psychiatric disorders that affect approximately one percent of the population worldwide. Several factors, such as disrupted-in-schizophrenia 1 (DISC1) [16], Neuregulin 1 [17], catechol-o-methyltransferase (COMP) [18,19,20], and BDNF [21], among others [22], have been associated with an increased risk to develop schizophrenia. However, the overall mechanism resulting in schizophrenia remains unclear. Schizophrenia is deeply related to neurodevelopmental alterations, and abnormalities may occur at a very restricted stage during brain development. With regard to the relationship of polySia with schizophrenia, abnormalities in polysialylation and polySia-expressing tissues have been reported [10,11]. Indeed, a decrease in polySia containing cells was observed in hippocampi from patients with schizophrenia [10]. Moreover, the small OB volume of patients with schizophrenia [23] is also a phenotype of NCAM-deficient mice [24]. Disturbance of the hippocampus anatomical organization is also involved in the etiology of schizophrenia [25], and observed in the ST8Sia2-deficient mice [26]. The phenotypes of ST8Sia2-knock out (KO) mice were well defined. ST8Sia2-KO mice showed normal migration of neural precursors in olfactory system, normal long-term potentiation (LTP) and long-term depression (LTD) in CA1, but showed abnormal mossy fiber projection in hippocampus, higher exploratory drive and reduced behavioral response to fear conditioning [26]. In addition, ST8Sia2-KO mice showed enlarged lateral ventricles and reduction of the thalamus. ST8Sia2-KO mice displayed impaired working memory and deficits in prepulse inhibition, anhedonic behavior and increased sensitivity to amphetamine-induced hyperlocomotion that are the characteristic phenotypes observed from SZ patients [13]. Therefore, ST8Sia2-KO mice are reported to be able to use as a model animal for analyzing SZ [13]. On the other hand, ST8Sia4-KO mice showed different phenotypes than those of ST8Sia2, abnormal LTP and LTD in CA1, and normal mossy fiber projection in hippocampus [27]. The polySia-deficient mice (ST8Sia2- and ST8Sia4-double KO mice) showed profound influences on the development of brain, and progressive hydrocephalus, postnatal growth retardation, and precocious death were observed [28]. Interestingly, NCAM-, ST8Sia2-, ST8Sia4-triple KO mice showed mild phenotypes with normal viability and body weight development, a slight and constant enlargement of the lateral ventricles, delaminated mossy fibers, abnormal migration of precursor neurons and smaller olfactory bulbs that were largely shown in NCAM-KO mice [28]. In humans, chromosome 15q26 where ST8SIA2/STX is localized was reported as a common susceptibility region for both schizophrenia and bipolar disorder in a genome scan of Eastern Quebec families [29]. Based on a genome-wide study, Arai et al. [12] also demonstrated an association between SNP-1 and -3 in the promoter region of the STX gene and schizophrenia in the Japanese population. Recently, we have demonstrated that a new function of polySia, that is, the molecule-binding property, is closely related to the polySia structure that appears to be strictly regulated by the biosynthetic enzymes, ST8SIA2/STX and ST8SIA4/PST [1,30]. We also demonstrated that polySia-NCAM biosynthesized by a mutated polysialyltransferase (ST8SIA2/STX), which was found in patients with schizophrenia, was impaired not only in the structure but also in the reservoir scaffold function [6,7,8]. It is thus suggested that a normal polySia expression is required for normal neuronal functions [1,30]. If this hypothesis holds true, it can be hypothesized that anti-schizophrenia reagents might improve these polySia properties. To demonstrate this hypothesis, the effects of chlorpromazine (CPZ), an old but clinically used anti-psychotic drug in schizophrenia [31], on the expression of polySia in cells and brain tissues was examined.

CPZ is a famous and first generation chemical medicine for schizophrenia [29,32]. It is effective on the positive symptoms of schizophrenia [32], which are the result of excess dopamine signal in the brain of patients with schizophrenia [33]. In particular, CPZ reduces the dopamine signal through inhibiting dopamine receptor 2 (D2R). The side effect of CPZ is the Parkinson’s disease (PD)-like behavior [31,32], indicating that dopamine signaling is reduced excessively. Interestingly, the expression of polySia is reported to be reduced in the schizophrenic brain [10,11] and to be upregulated in PD [34]. In addition, reduction of polySia on NCAM in human neuroblastoma cells influenced the dopamine signaling [8,35]. However, the effects of CPZ on polySia expression remain unknown.

In this study, we analyzed the changes of cell surface polySia using human neuroblastoma cells, which are used for investigating human neuronal cell behavior and for drug screening [36,37]. Our findings demonstrated that polySia is not regulated by gene expression, but by the endocytosis/exocytosis system.

## 2. Results

### 2.1. Effects of CPZ on PolySia Expression Using IMR-32 Human Neuroblastoma Cells

**Figure 1:**
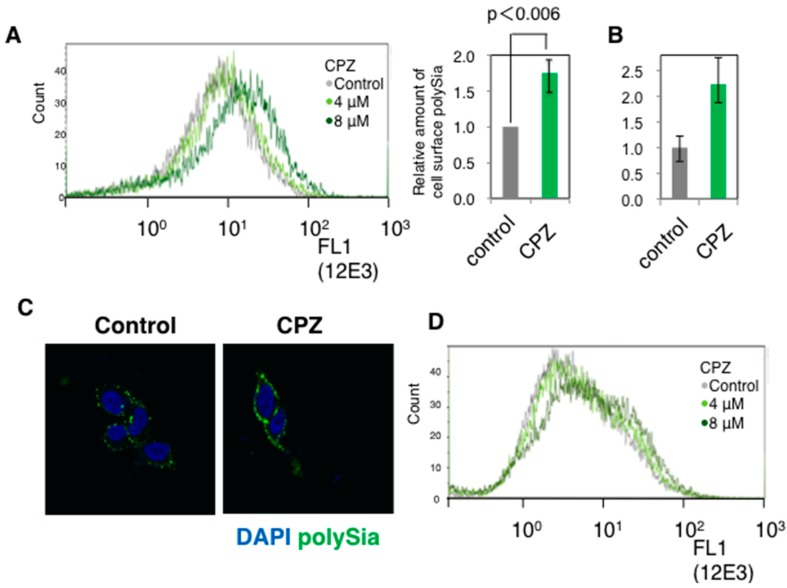
PolySia expression on the cell surface of human neural cells. (**A**) The polySia expression on IMR-32 cells before and after CPZ treatment using anti-polySia antibody was analyzed by flow cytometry. The cells were treated with 0, 4, and 8 μM of CPZ and subsequently incubated with anti-polySia antibody. PolySia expression was observed using Alexa-488 (FL1) labeled anti-mouse IgM antibody. The right panel shows the relative amounts of cell surface-polySia evaluated by mean fluorescence intensity (MFI) (*n* = 3). The MFI of control (0 μM CPZ) was set to 1.0; (**B**) Chemical evaluation of the polySia amounts on the cell surface before and after treatment with CPZ. PolySia derived from the cells (2.5 × 10^5^) treated with or without CPZ (8 μM) was cleaved by Endo-N. Released oligoSia from polySia chain was labeled with 1,2-diamino-4,5-methylenedioxybenzene (DMB) and measured by HPLC analysis. The experiments were performed and the amount of polySia before CPZ treatment was set to 1.0. SQ (*n* = 2), CPZ (*n* = 3); (**C**) Immunocytostaining of IMR-32 cells. The polySia structure (Green) was immunostained with anti-polySia antibody (12E3) and Alexa488-labeled anti-mouse IgM antibody. The nucleus was stained with 4′,6-diamidino-2-phenylindole (DAPI) (Blue). The cells were visualized using confocal microscopy; (**D**) The polySia expression on cell surface of SK-N-SH cells before and after CPZ treatment using anti-polySia antibody was analyzed by flow cytometry. The cells were treated with 0, 4, and 8 μM of CPZ and subsequently incubated with anti-polySia antibody (12E3). PolySia expression was observed using Alexa-488 (FL1) labeled anti-mouse IgM antibody.

First, we examined the polySia-expression before and after administering CPZ on the cell surface of cells of the human neuroblastoma cell line IMR-32, which is used as a model human neuronal cell line [36,37]. As evidenced by the anti-polySia antibody 12E3 immunostaining, PolySia-NCAM significantly increased on the cell surface of IMR-32 following the addition of CPZ (Figure 1A). Then we analyzed the amount of cell surface polySia released after endo-*N*-acylneuraminidase (Endo-N) treatment and found that the amount of polySia on the cell surface was increased 2.2 times after CPZ treatment (Figure 1B). The cell surface staining of polySia was also confirmed under microscopy (Figure 1C). The dotted staining of 12E3 was a characteristic of the IMR-32 cell surface and was up-regulated after CPZ addition. The increased expression of polySia-NCAM on the cell surface after CPZ addition was also observed in SK-N-SH, which is another human neuroblastoma cell line (Figure 1D). These data indicate that CPZ upregulated the cell surface polySia-expression in human neuroblastoma cells.

**Figure 2:**
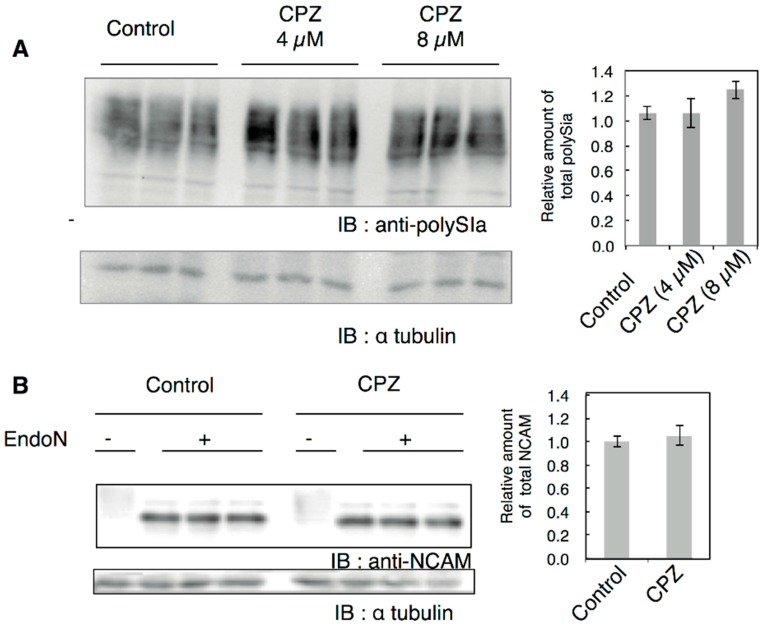
PolySia expression of the cell from human neural cells. (**A**) The polySia expression of IMR-32 cells before and after CPZ treatment using anti-polySia antibody was analyzed by SDS-PAGE/Western-blotting. The cells were treated with 0, 4 and 8 µM of CPZ and then homogenized. The PolySia expression of total cell homogenate was analyzed using anti-polySia antibody (12E3). The left panel shows the polySia staining (anti-polySia) and anti-tubulin staining (anti-tubulin). The right shows the relative amount of total polySia expression. The control polySia staining divided with tubulin staining was set to 1.0 (*n* = 3); (**B**) The NCAM expression of IMR-32 cells before (control) and after CPZ treatment (CPZ, 8 μM) using anti-NCAM antibody was analyzed by SDS-PAGE/Western-blotting. The homogenates before and after Endo-N treatment were analyzed. The right shows the relative amount of total NCAM expression. The control NCAM staining divided with tubulin staining was set to 1.0 (*n* = 3).

Subsequently, we analyzed the change in total polySia-containing NCAM using cell homogenates. The amount of total polySia was not significantly changed based on the Western blotting analysis using anti-polySia antibody (Figure 2A). In order to evaluate changes in NCAM, we treated the cell homogenates with Endo-N [38] to cleave the polySia structure for the evaluation of whole NCAM (Figure 2B, Endo-N). After treating the homogenates with Endo-N, NCAM-140 was exposed to reveal that there were no changes in the amount of NCAM before and after treatment with CPZ (Figure 2B). As NCAM was not stained in the intact cell homogenates, it appeared to be polysialylated almost entirely.

**Figure 3:**
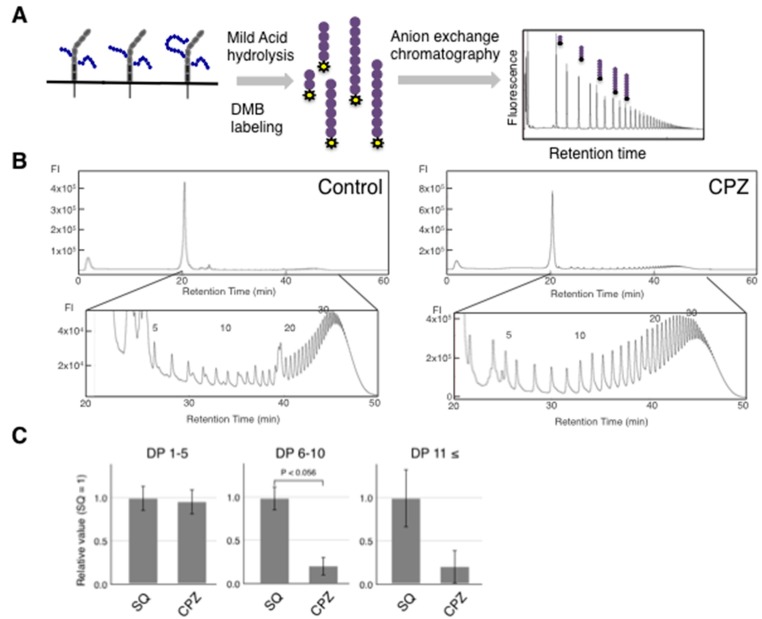
Characterization of polySia-NCAM of IMR-32 cells. (**A**) Scheme of the mild-acid hydrolysis (MH)-fluorometric anion-exchange chromatography (MH-FAEC) analysis. Samples (polySia-NCAM in homogenates: Purple circle indicates sialic acid) were hydrolyzed under mild acid conditions and were then immediately labeled with the α-keto acid-specific fluorometric reagent DMB (yellow). The labeled oligo/polySia chains were subjected to anion-exchange chromatography. The elution pattern of colominic acid is shown. Using this method, it is possible to estimate the chain length (degree of polymerization, DP) of polySia based on peak number; (**B**) MH-FAEC analysis of polySia-NCAM in IMR-32. Samples were subjected to mild-acid hydrolysis, and released oligo/polySia were derivatized with DMB. Labeled samples were loaded onto a DNApac PA-100 anion-exchange column and were eluted with 2 mM Tris-HCl (pH 8.0), followed by a NaCl gradient (0–15 min, 0 M; 15–60 min, 0.6 M) at a flow rate of 1 mL/min. The elution profile was monitored with a fluorescence detector by excitation at 373 nm and emission at 448 nm as described in materials and methods; (**C**) Quantitative analysis of oligo/polySia. PolySia chains derived from whole cell lysate of IMR32 incubated with CPZ (*n* = 3) were qualified by the MH-FAEC. The oligo/polySia with DP 1~5, 5~10, greater than 11 were qualified.

To understand the change of the quality of polySia-NCAM, we analyzed the DP of polySia-NCAM in IMR-32 chemically before and after CPZ treatment using mild acid hydrolysis-fluorometric anion exchange chromatography analysis (MH-FAEC) [1,39]. The whole cell lysate before and after addition of CPZ was treated with mild acid and released oligo/polySia chains were labeled with 1,2-diamino-3,4-methylenedioxybenzene (DMB) (Figure 3A). We analyzed the labeled oligo/polySia using an anion-exchange chromatography to evaluate the DP of polySia; the DP composition is shown in Figure 3C. The maximum DPs observed were the same between treated and untreated cells (Figure 3B). We found that the amounts of oligo/polySia derived from whole cell homogenates with DP greater than 6 were dramatically decreased after incubation with CPZ (Figure 3C).

**Figure 4:**
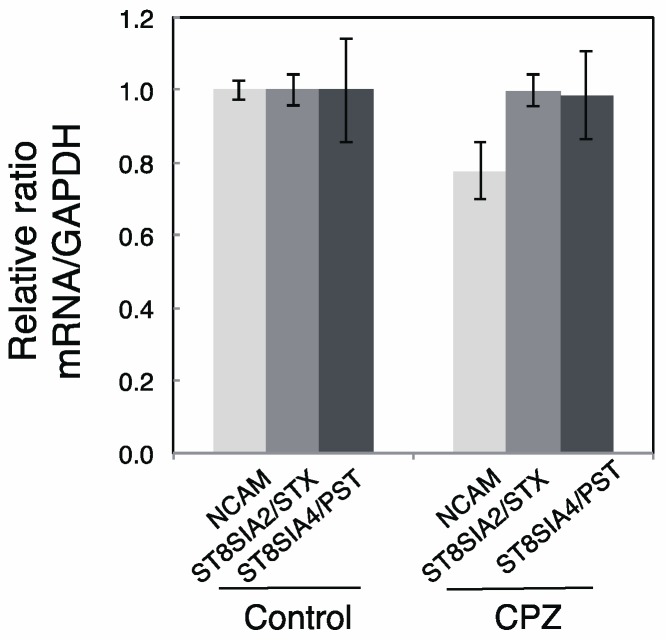
The change of mRNA for ST8SIA2/STX, ST8SIA4/PST, and NCAM before and after CPZ treatment. RT-PCR was performed using IMR-32 cells before and after treatment with CPZ. Aliquots of the PCR products amplified were analyzed for the expression of the mRNAs for ST8SIA2 (30 cycles), ST8SIA4 (30 cycles), NCAM (30 cycles), and actin (20 cycles) on 1% or 2% agarose gel electrophoresis. The amounts of mRNA for ST8SIA2, ST8SIA4, or NCAM were divided with that for actin. The control amounts (without CPZ incubation) were set to 1.0 (*n* = 3).

To confirm the change of the gene expressions after adding CPZ, we performed semi-quantitative PCR analyses using the templates derived from PBS and CPZ incubated IMR-32 cells. The mRNA expressions for NCAM, ST8SIA2/STX, and ST8SIA4/PST were not changed significantly (Figure 4; Control versus CPZ). These results are consistent with the unchanged amounts of polySia-NCAM and NCAM after CPZ addition.

**Figure 5:**
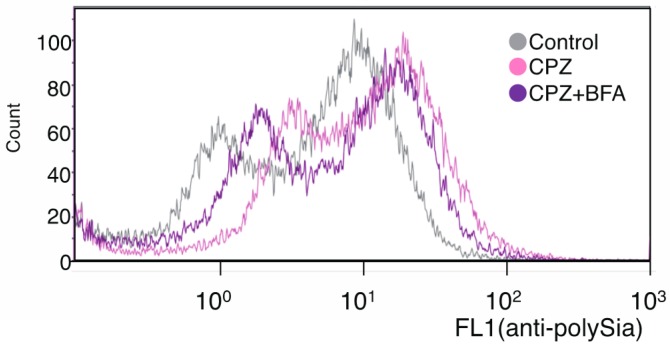
Endocytotic regulation of polySia-expression on cell surface of neural cells. PolySia expression on the cell surface of IMR-32 cells with or without Brefeldin A (BFA) under CPZ conditions were analyzed using anti-polySia antibody by flow cytometry.

Therefore, the up-regulation of cell surface polySia was considered to be due to the accelerated exocytosis of polySia-NCAM on the cell surface or inhibition of endocytosis. Although it is known that cell surface NCAM is regulated by a recycling system [40,41], it remains unknown whether polySia-NCAM on cell surface is also regulated by such a mechanism. To confirm that polySia-NCAM on cell surface is regulated by a recycling system, we used a specific reagent, Brefeldin A (BFA), which inhibits the recycling pathway [42]. BFA was added to the CPZ-treated IMR-32, and the cell surface polySia-NCAM was analyzed by flow cytometry. After incubation with BFA, the cell surface polySia was down-regulated after a 30-min BFA incubation (Figure 5), compared with incubation with only CPZ. This indicates that the CPZ-induced up-regulation of polySia-NCAM expression on the cell surface is regulated in part via the recycling system.

### 2.2. Effects of CPZ on PolySia Expression Using Mouse Brains

**Figure 6:**
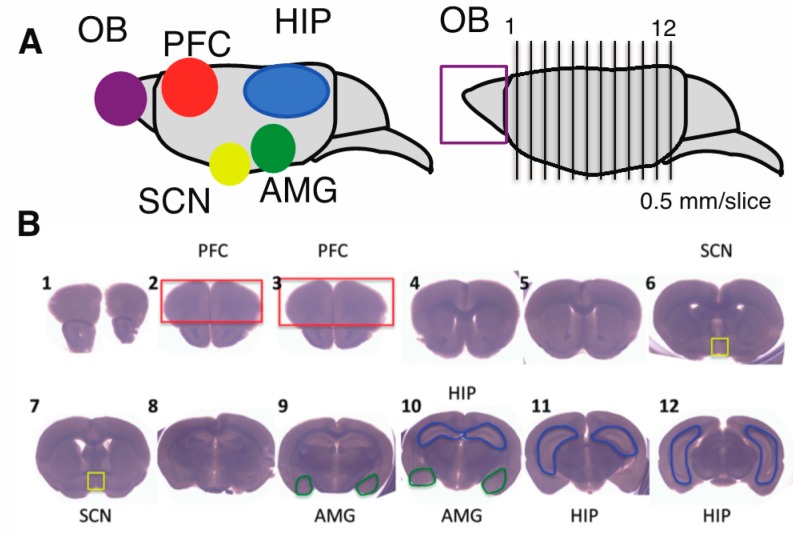
Sample preparation from mouse brains. (**A**) Brain parts for experiments. The 5 brain parts (OB: Olfactory bulb, HIP: Hippocampus, AMG: Amygdala, SCN and PFC: Prefrontal cortex) for the experiments were shown in the image of brain (**left**). Brain sections (**right**). Dissected brain was separated into OB and other part. Other brain part was cut into 12 sections (0.05 mm/section); (**B**) The 12 sections from mouse brain (**A**). From these sections, four parts (PFC: Red, SCN: Yellow, HIP: Blue, and AMG: Green) were dissected and homogenized for further experiments.

Our findings suggest clearly that CPZ had the effect on the up-regulated expression of polySia-NCAM on cell surface in human neural cells. In brains from patients with schizophrenia, it has been reported that polySia expression was down-regulated in the prefrontal cortex (PFC) [11]. Although the total amounts of polySia-NCAM were not changed after CPZ in human neuroblastoma cells, we predicted that, since CPZ is an effective treatment for schizophrenia, polySia expression, especially in the PFC, could be recovered after CPZ administration. Then we used mice to understand whether polySia expression is changed after CPZ administration in specific brain regions, including OB, HIP, amygdala (AMG), suprachiasmatic nucleus (SCN), and PFC (Figure 6A,B).

**Figure 7:**
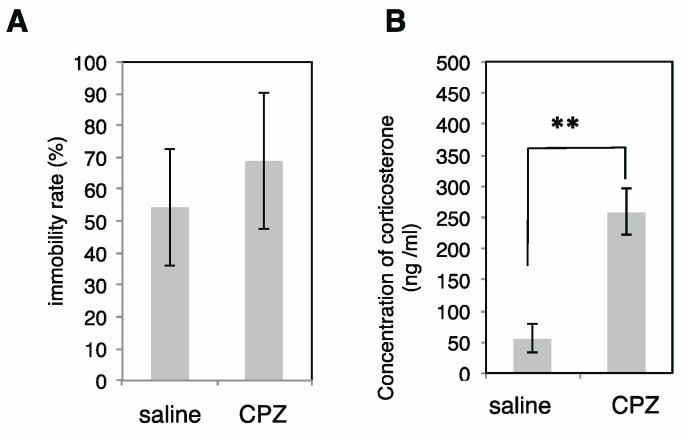
Diagnosis of mice treated with or without CPZ. (**A**) Tail suspension test of mice. Mice were treated with or without CPZ as described in material and methods and Immobility rate by TST was measured (*n* = 5); (**B**) The concentration of the corticosterone. The concentration of the corticosterone in serum derived from mice after administration of saline or CPZ was analyzed (*n* = 5). ** indicates *p* < 0.005.

We injected CPZ via intraperitoneal (i.p.) administration into mice and subsequently analyzed the immobility rate. As shown in Figure 7A, no changes were observed in the immobility rate evaluated using the tail suspension test (TST), indicating that CPZ does not influence the depression state in mice. This is consistent with a previous report [43]. We also observed a significant increase in the concentration of serum corticosterone following CPZ administration (Figure 7B).

**Figure 8:**
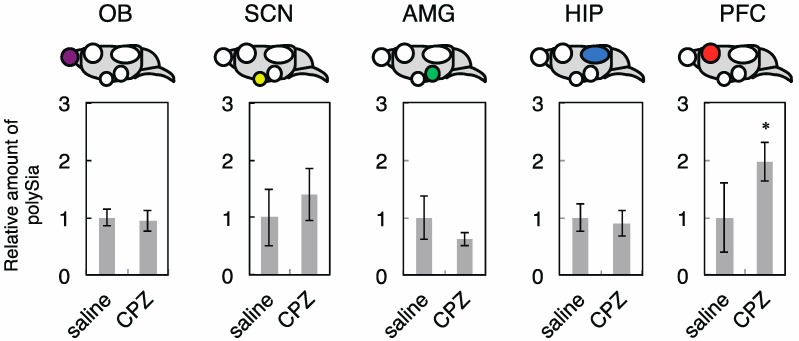
PolySia expression in five regions from mice brain treated with or without CPZ. The polySia expression in five regions after administration with saline and CPZ evaluated by anti-polySia antibody (735) staining were shown (*n* = 5). The polySia expression after saline administration was set to 1.0. * indicates *p* < 0.05.

Subsequently, we dissected the five brain regions (Figure 6A,B) and analyzed the amount of polySia-NCAM in each in saline- and CPZ- treated mice. As shown in Figure 8, the expression of polySia-NCAM was not changed in OB, HIP, AMG, and SCN after CPZ injections.

**Figure 9:**
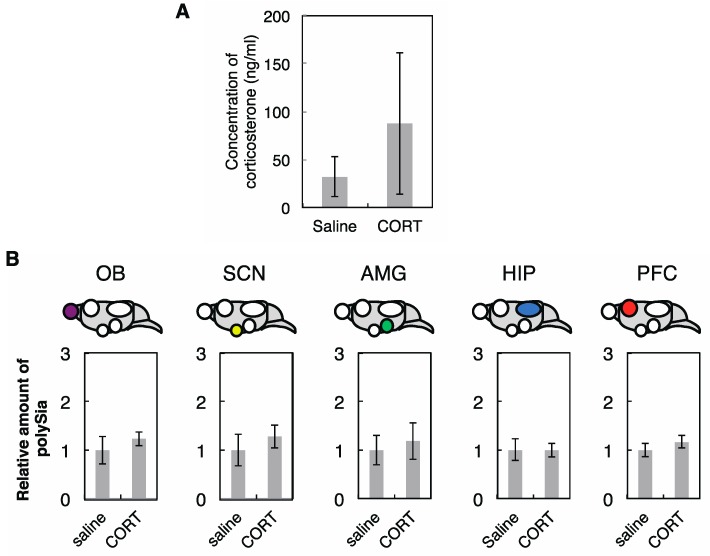
Diagnosis of mice treated with or without corticosterone. (**A**) The concentration of the corticosterone. The concentrations of the corticosterone in serum derived from mice after injection with saline or corticosterone were analyzed (*n* = 4); (**B**) PolySia expression after corticosterone treatment. The polySia expressions in five regions after administration with saline and corticosterone evaluated by anti-polySia antibody (735) staining were shown (*n* = 5). The polySia expression after saline administration was set to 1.0. * indicates *p* < 0.05.

These results were consistent with the results observed using IMR-32 cells (Figure 2). However, in PFC, the expression of polySia-NCAM was clearly up-regulated as evidenced by the anti-polySia antibody 735, which is typically used for the polySia-immunostaining as a polySia probe in the brain. Given that the corticosterone concentration increased significantly after CPZ injection (Figure 7B), we analyzed the effects of corticosterone on the polySia expression in the five brain regions. We administrated corticosterone and measured the concentration of corticosterone in serum to confirm the up-regulation of the corticosterone in serum (Figure 9A).

As shown in Figure 9B, the expressions of polySia in the five brain regions remained unchanged in the presence of corticosterone, indicating that up-regulated expression of polySia after CPZ treatment might be due to the effects of CPZ, but not corticosterone.

## 3. Discussion

The decreased expression of polySia-NCAM in PFC and decreased number of polySia-containing cells in HIP derived from patients with schizophrenia were observed previously in comparison with individuals without schizophrenia [10,11]. Based on the biochemical analyses, we confirmed that the SNP-7 of ST8SIA2, which is a point mutation that was observed in a patient with schizophrenia, impaired polySia-NCAM both quantitatively and qualitatively [6,7,8]. The impaired polySia structure was shown to lead to the impairment of the polySia functions [6,7,8]. Based on these results, recently, we hypothesized that the expression of polySia is highly regulated and the abnormal expression of polySia leads to a high risk of disease [1,30]. Therefore, we focused on the CPZ, a drug used in schizophrenia, to alter the expression of polySia in cells and brain tissues.

**Figure 10:**
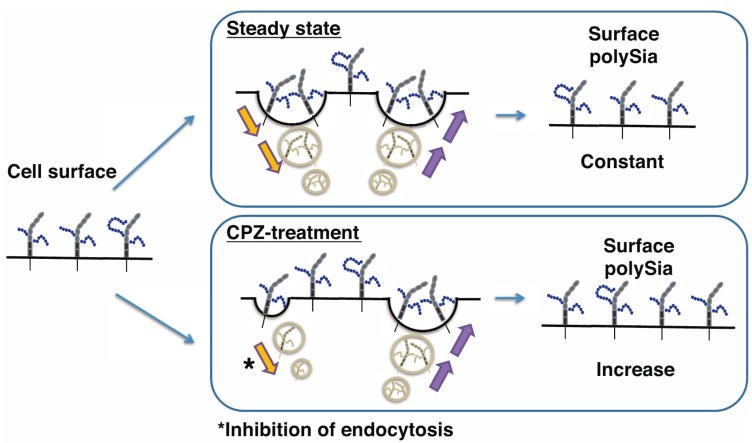
PolySia expression on the cell surface of human neuroblastoma cells. At the cellular level, cell surface polySia expression is highly regulated via regulated balance between endocytosis and exocytosis. If the balance is changed such as drugs or other environmental factors, polySia expression is changed. In the case of CPZ, the cell surface polySia expression is increased probably due to the inhibition of the endocytosis. * indicates the inhibition of endocytosis.

Since the decreased expression observed in section staining using anti-polySia antibody indicates the decreased expression of total polySia-NCAM, but not the case with polySia-NCAM expression on the cell surface, we predicted that the polySia expression is up-regulated after CPZ treatment. As a human neural cell model, we used human neuroblastoma cells, which have been widely used as model cells for understanding neural function or for drug screening [36,37]. After adding CPZ, cell surface polySia-NCAM was increased (Figure 1); however, surprisingly, no change of total polySia-NCAM was observed (Figure 2). This was the same for the polySia-NCAM related gene expression (Figure 4), indicating that CPZ influenced only the cell surface polySia-expression in IMR-32 cells. Since CPZ is also used as an inhibitor of clathrin-related endocytosis [44,45,46], we chose BFA to inhibit secretory pathway. We found that the addition of BFA with CPZ decreased the effect of CPZ on the polySia expression on the cell surface, suggesting that the regulated clathrin-related endocytosis system was functional for polySia-expression. Previously, we found that the iSNP of the ST8SIA2 gene, which has been reported to be associated with bipolar disorder, results in an increase in mRNA of the ST8SIA2 gene, ST8SIA2 and their product, and polySia-NCAM as compared with controls; however, the cell surface expression of polySia was not significantly changed [47]. These results also suggest that the amounts of the cell surface polySia-NCAMs are highly regulated and that not all polySia-NCAMs are present on the cell surface; indeed, some polySia-NCAMs may be in intracellular vesicles. Although non-polysialylated NCAMs (NCAM-140 and NCAM-180) have been shown to be regulated via recycling system [38], our present study is the first to demonstrate the recycling of polySia-NCAM in human neuroblastoma cells (Figure 10).

It was also clearly demonstrafiguted in this study that, although the total polySia was not changed in IMR cells, the quality, especially the DP, was changed after CPZ treatment. Based on reduced proportions of longer to shorter DPs in polySia-NCAM derived from CPZ-treated cells, the polySia DP was decreased. As the genes for polysialyltransferases and NCAM were not significantly changed (Figure 4), a degradation of DP might have occurred in the presence of CPZ. Rapid degradation of polySia via the exocytosed sialidase Neu1 through exosome secretion has been previously shown [48]. Therefore, lower polySia DP emerged might be due to Neu1. In addition, the secretion of lysosomal components via exocytosis has been reported to be inhibited by CPZ [49]. The pH change toward acidic conditions via secretion of lysosomal components might lead to the degradation of polySia, whose structure is highly sensitive to the acidic pH [50]. As the CPZ inhibited the recycling and secretory pathway of the molecules, even at lower concentrations, the CPZ, as an anti-schizophrenic agent, might have efficacy toward clathrin-mediated endocytosis that regulates functions of the molecules such as polySia-NCAM; although CPZ is a well-known medicine for the dopamine D2 receptor antagonist [31,32]. Therefore, the balance between polySia on the cell surface and total polySia is highly regulated (Figure 10) and might be a key phenomenon in regulating the state of the diseases.

To understand the effects of CPZ on polySia expression in vivo, we used a mouse model. After administration of the CPZ into mice, as expected from the results using human neuroblastoma cells (Figure 1 and Figure 2), the total polySia-expressions in OB, HIP, AMG, and SCN were not changed significantly (Figure 8). Analyzing the expression of polySia using homogenates or tissue staining has the limitation of discriminating the amounts between cell surface polySia and total polySia. Therefore, caution should be considered when analyzing brain sections immunostaining using anti-polySia antibody as it might reflect the total polySia. However, interestingly, we found that the total amount of polySia-NCAM evaluated by anti-polySia antibody 735 was up-regulated in the PFC in mice (Figure 8, PFC); however, the underlying mechanism is still unknown. We confirmed that the high concentrations of corticosterone were not the cause of the up-regulated expression of polySia because no change of the polySia expression was observed after administration of corticosterone (Figure 9). Therefore, it is suggested that the decreased expression of polySia from brains of patients with schizophrenia reported in PFC [11] might be improved via CPZ administration. In other words, up-regulation of polySia by CPZ might contribute to the cure of symptoms of schizophrenia. Based on the results obtained from mice brain tissues, it is important to note that the effects of polySia expression via CPZ were brain region-specific and environmental factors such as CPZ influenced particular brain regions. The mice showed no improvement of the immobilization rate on tail suspension test (TST) following CPZ treatment, indicating that CPZ is not effective for depression. Recently, it has been shown that CPZ administration leads to the high serum corticosterone concentrations in mice. Based on our results, high corticosterone concentrations did not influence the total polySia expression in neither of the five brain tissues, indicating that corticosterone, which is a marker of stress, might not influence the total polySia-expression of the OB and PFC in mice.

## 4. Materials and Methods

### 4.1. Materials

Human neuroblastoma IMR-32 and SK-N-SH were purchased from Japanese Collection of Research Bioresources (JCRB) cell bank (Kobe, Japan). Enhanced chemiluminescence (ECL) reagents were obtained from GE healthcare (Piscataway, NJ, USA). Eagle’s Minimum Essential Medium (MEM) and Minimum Essential Medium Eagle Alpha Modification (α-MEM), bovine serum albumin, and phenylmethylsulfonyl fluoride (PMSF) were purchased from Sigma-Aldrich (St. Louis, MO, USA). Mouse anti-NCAM antibody (123C3) was purchased from Abcam (Tokyo, Japan). Synthetic oligonucleotide primers were obtained from Rikaken (Nagoya, Japan). Molecular weight marker and bicinchoninic acid (BCA) assay kit were purchased from Bio-rad (Hercules, CA, USA). The 12E3 antibody, which was purified before use, was from Tatsunori Seki (Tokyo Medical University) [51]. Anti-polySia recombinant antibody, 735scFv was prepared as described previously [52]. Endo-*N*-acylneurminidase was generously gifted from Frederic F Troy II (University of California, Davis, CA, USA) [38] and subsequently purified. Polyvinylidene difluoride (PVDF) membrane (Immobilon P) was a product of Millipore (Bedford, MA, USA). DMB was purchased from Dojindo (Kumamoto, Japan). Colominic acid (polyNeu5Ac) and Handy-ODS were from Wako (Osaka, Japan). Peroxidase-conjugated goat anti-mouse (IgG + IgM) was purchased from American Qualex (San Clemente, CA, USA).

### 4.2. Cell Culture

Human neuroblastoma IMR-32 cells were cultured in Eagle’s Minimum Essential Medium (Sigma) supplemented with 1% Non-Essential Amino Acids (NEAA), 0.5 mg/mL of streptomycin sulfate, 100 units/mL of penicillin G, and 10% fetal bovine serum in a 5% CO_2_ and 95% air humidified atmosphere at 37 °C. Human neuroblastoma SK-N-SH cells were cultured in α-MEM (Sigma, St. Louis, MO, USA) supplemented with 0.5 mg/mL streptomycin sulfate, 100 units/mL of penicillin G, and 10% fetal bovine serum in a 5% CO_2_ and 95% air humidified atmosphere at 37 °C.

### 4.3. Treatment of Chlorpromazine (CPZ) Using Neural Cells

IMR-32 or SK-N-SH (1.5 × 10^6^) cells were plated on a 9-cm dish and incubated for 24 h in MEM (10) or αMEM (10). After rinsing with phosphate buffered saline (PBS), cells were incubated with either MEM (10) or αMEM (10) containing 0, 4, or 8 μM CPZ and were incubated for 1 h or 3 day. Subsequently, cells were rinsed with PBS and divided into three groups. One group was used for sodium dodecyl sulfate polyacrylamide gel electrophoresis (SDS-PAGE)/Western-blotting for whole cell analysis, one for flowcytometer for cell surface analysis, and one for Reverse transcription-polymerase chain reaction (RT-PCR) to analyze gene expression.

### 4.4. SDS-PAGE and Western Blotting

Cells were homogenized with PBS containing 1% triton X-100, protease inhibitor cocktail (1 μg/mL of aprotinin, leupeptin, and pepstatin, 2 μg/mL of antipain), and 5 mM EDTA and incubated on ice for 1 h. Homogenates were centrifuged and the protein concentration of the supernatants was evaluated by the bicinchoninic acid (BCA) assay. Samples were dissolved in Laemmli buffer containing 5% mercaptoethanol and were subsequently incubated at 60 °C for 20 min for glycans or 100 °C for 3 min for proteins. The denatured samples were then electrophoresed on 7% polyacrylamide gels and electroblotted onto PVDF membranes using a semi-dry blotting apparatus. Following the transfer, PVDF membranes were blocked at 25 °C for 1 h with PBS containing 0.05% Tween 20 (PBST) and 1% skim milk for NCAM staining or bovine serum albumin for polySia staining. The membranes were then incubated overnight with the primary antibody, 12E3 (10 μg/mL), or anti-NCAM antibody (4 μg/mL) at 4 °C overnight. As the secondary antibody, peroxidase-conjugated anti-mouse IgG + IgM (0.4 μg/mL; American Qualex) was used at 37 °C for 45 min, and stained bands were visualized with chemiluminescent reagents (GE Healthcare). For staining with anti-NCAM antibody, cell homogenates were treated with Endo-N (0.9 mU in 20 mM Tris-HCl, pH 7.5 for 20 h) to cleave the polySia structure.

### 4.5. Flow Cytometry Analysis

Cells were collected with a cell scraper and rinsed with PBS. Subsequently, cells were incubated with the monoclonal antibody mAb 12E3 (10 µg/mL) at 4 °C for 1 h and then rinsed twice with PBS. The cells were then incubated with Alexa-labeled anti-mouse IgG or IgM (2 µg/mL) at 4 °C for 1 h. After rinsing cells twice with PBS, cell surface staining was measured using a flow cytometer (Gallios, BeckmanCoulter, Brea, CA USA) and the collected data was analyzed with the Kaluza software (Version1, BeckmanCoulter).

### 4.6. Reverse Transcription-Polymerase Chain Reaction (RT-PCR)

Total RNA was prepared from IMR-32 cells using TRIZOL (Molecular Research Center, Inc., Cincinnati, OH, USA) as previously described [9]. Random-primed cDNA (~50 ng) and the following specific primers were used for PCR: *NCAM*-s: 5′-CTGGAAACACAAAGGCCGAG-3′; NCAM-as: 5′-GATAGCTGGCAGAGGGGGTG3′; ST8SIA2/STX-s: 5′-CTGGATGCTGGCCGCGCTCA-3′ ; ST8SIA2/STX-as: 5′-CCATCGCACTGGCCGACAGT-3′; ST8SIA4/PST-s: 5′-ATGCGCTCCATTAGGAAGAG-3′; *ST8SIA4/PST*-as: 5′-AATGGCATTCTGTGAGGGCTT-3′; *Actin*-s: 5′-CTGGAGAAGAGCTACGAGCTGC-3′; and *Actin*-as: 5′-CGTGGCACTTCATGATGGA-3′. The mRNA quantifications of ST8SIA2/STX, ST8SIA4/*PST*, NCAM, and actin were performed by PCR (30 cycles, 30 cycles, 25 cycles, and 20 cycles, respectively).

### 4.7. Mild Acid Hydrolysis-Fluorometric Anion-Exchange Chromatography Analysis (MH-FAEC)

Samples (1.0 mg protein as BSA) in 200 μL of TFA (0.005 N) were added to 200 μL DMB solution and incubated at 50 °C for 1 h. Subsequently, cold ethanol was added to the samples (final concentration, 95%) and stood at −80 °C for 2 h. After centrifugation, the supernatants were dried in Speed Vac. The dried samples were dissolved in water and NaOH was added to a final concentration of 0.1 N and incubated at 37 °C to remove lactonization. After neutralization with HCl (1 N), the samples were diluted and subjected to an anion-exchange chromatography column (DNApac PA-100, 4 mm × 250 mm, DIONEX) and separated on a JASCO HPLC system. The sample was loaded on a column and eluted with 2 mM Tris-HCl (pH 8.0), followed by NaCl gradient (0–15 min, 0 M; 15–60 min, → 0.6 M) in 2 mM Tris-HCl (pH 8.0). The flow rate was 1 mL/min and fractions were monitored with a fluorescent detector (Em 373nm, Ex 448 nm, FP-2020, JASCO, Tokyo, Japan) [39].

### 4.8. Brefeldin Treatment

Cells were treated with or without brefeldin A (final concentration, 8 μM) and incubated for 1 h at 37 °C. Subsequently, the cells were analyzed using anti-polySia antibody by flow cytometry [9].

### 4.9. Cell Staining

Cells were fixed with 4% paraformaldehyde at 25 °C for 8 min after rinsing with PBS. The fixed and rinsed cells were blocked with 2% BSA in PBS and then incubated with 12E3 (mouse IgM, 30 µg/mL) at 4 °C for 1 h. After rinsing with PBS, the cells were incubated with Alexa488-conjugated anti-mouse IgM antibody (2 µg/mL; Invitrogen, Carlsbad, CA, USA). The cells were rinsed three times with PBS and then incubated with 4′,6-diamidino-2-phenylindole (DAPI, 1 µg/mL) at 37 °C for 10 min, followed by a single rinse with PBS. Cells were then observed using a confocal scanning fluorescent microscope (Olympus, Tokyo, Japan).

### 4.10. Measurement of PolySia on the Cell Surface

Cells were incubated in the 6-well plate (2.5 × 10^5^) and added CPZ (final concentration 8 μM). After 1 h incubation, cells were washed with PBS and added EndoN solution (final concentration 0.3 mU/mL) to evaluate the amount of polySia on cell surface [53]. After incubation at 37 °C for 30 min, supernatant was collected and released oligoSia was pooled as filtrates using VIVASPIN (cut off: 10kDa). Collected oligoSia fractions were derivatized with DMB. Labeled oligoSia-DMB (diSia, triSia, tetraSia) eluted before monoSia-DMB was measured using ODS column (Wako, Osaka, Japan) (Acetonitrile/Methanol/Water = 9:7:87).

### 4.11. Animals and Ethics Statement

Ten mice (3 months old, Chubu Kagaku Shizai, Nagoya, Japan) were used in this study (five mice in each of the two experimental groups). Mice were housed in a controlled environment (23 ± 2 °C and 50 ± 10% humidity, 12:12 light/dark cycle) with food and water available ad libitum. Mice were allowed to acclimatize to our facilities for one week prior to the start of the experiments. All procedures were approved by the Animal Care and Use Committee of Nagoya University (No.2016022506, 28 March 2016). Every effort was made to minimize the number of animals used and their suffering.

### 4.12. Drug Treatment

CPZ (Tokyo Chemical Industry, Japan) was dissolved at a concentration of 0.5 mg/mL in isotonic (0.9% NaCl) saline solution immediately before use. Mice were divided to two groups based on the treatment, i.e., CPZ and control groups [54]. In the CPZ group, mice were injected with CPZ (2 mg/kg, i.p.), while mice in the control group were administered the same volume of saline.

### 4.13. Preparation of the Brain Sample

One hour after CPZ treatment, mice were sacrificed by CO_2_ administration. The cerebrum was collected by surgery and rinsed with PBS. The OB was collected first. The remaining brain was embedded in 1% agarose/PBS and immediately sliced into 500-μm sections. The PFC, SCN, AMG, and HIP were collected from the sections. These regions were homogenized with PBS containing 1% Triton-X100, 1 mM PMSF, 1 μg/mL leupeptin, 2 μg/mL antipain, 10 μg/mL benzamidine, 1 μg/mL pepstain, 1 μg/mL aprotinin, 1 mM EDTA, 50 mM NaF, 10 mM β-Glycerophosphate, 10 mM Sodium Pyrophosphate dehydrate, and 1 mM sodium *o*-vanadate. The homogenates were incubated on ice for 1 h and centrifuged at 10,000 rpm for 15 min at 4 °C. The supernatant was used after measurement of protein concentration by the BCA method. The homogenates were analyzed as described in SDS-PAGE/Western-blotting.

### 4.14. Data Processing

All values are expressed as the mean ± SD (*n* is indicated) and *p*-values were evaluated by the student *t*-test.

## 5. Conclusions

In conclusion, the polySia-NCAM in human neuroblastoma cells is regulated by the recycling system and CPZ influenced the up-regulated expression of polySia-NCAM on the cell surface. In contrast, total polySia-NCAM expression was up-regulated only in mice PFC. Therefore, CPZ may be effective in regulating the expression of polySia-NCAM probably via a recycling mechanism.

## Abbreviations

BD	bipolar disorder
BDNF	brain-derived neurotrophic factor
FGF2	fibroblast growth factor-2
HIP	hippocampus
NCAM	neural cell adhesion molecule
SZ	schizophrenia
